# Remote Ischaemic Preconditioning Accelerates Brain to Blood Glutamate Efflux via EAATs-mediated Transport

**DOI:** 10.1007/s11064-023-04002-x

**Published:** 2023-08-02

**Authors:** Jana Končekova, Klaudia Kotorova, Miroslav Gottlieb, Martin Bona, Petra Bonova

**Affiliations:** 1 Institute of Neurobiology, Biomedical Research Center of the Slovak Academy of Sciences, Soltesovej 4-6, Košice, 040 01 Slovak Republic; 2 Department of Medical Physiology, Faculty of Medicine, University of Pavol Jozef Safarik, Košice, 040 01 Slovak Republic

**Keywords:** Remote ischaemic preconditioning, Glutamate, Glutathione, Stroke, Excitatory amino acid transporters

## Abstract

Remote ischaemic conditioning (RIC) becomes an attractive strategy for the endogenous stimulation of mechanisms protecting neurons against ischaemia. Although the processes underlying the RIC are not clearly understood, the homeostasis of glutamate seems to play an important role. The present study is focused on the investigation of the brain to blood efflux of glutamate in a condition mimicking ischaemia-mediated excitotoxicity and remote ischaemic preconditioning (RIPC). The animals were pre-treated with a hind-limb tourniquet one hour before the intraventricular administration of glutamate and its release was monitored as the concentration of glutamate/glutathione in blood and liquor for up to 1 h. The transport mediated by excitatory amino acid transporters (EAATs) was verified by their inhibition with Evans Blue intraventricular co-administration. RIPC mediated the efflux of glutamate exceeding from CSF to blood in the very early stage of intoxication. As a consequence, the blood level of glutamate rose in a moment. EAATs inhibition confirmed the active role of glutamate transporters in this process. In the blood, elevated levels of glutamate served as a relevant source of antioxidant glutathione for circulating cells in RIPC-treated individuals. All of those RIPC-mediated recoveries in processes of glutamate homeostasis reflect the improvement of oxidative stress, suggesting glutamate-accelerated detoxication to be one of the key mechanisms of RIPC-mediated neuroprotection.

## Background

Stroke is the second-leading cause of death and a major cause of disability worldwide. Although much of the global burden of stroke measured in proportion to mortality is allocated to haemorrhagic stroke, ischaemic stroke is an equally fatal event, often ending in death (reviewed in [1]). The first aid for an ischaemic stroke patient is the administration of a thrombolytic drug; however, is time limited and with a frequent complication of haemorrhagic turnover. Therefore, any additional live-saving intervention would be highly valuable. Currently, great attention is being paid to the outcomes of several clinical trials testing alternative non-pharmacological interventions for stroke, which is known as remote ischaemic conditioning.

Remote ischaemic conditioning (RIC) refers to a process whereby periods of intermittent ischaemia, typically via the cyclical application of a blood pressure cuff to a limb above systolic pressure, confer systemic protection against ischaemia in spatially distinct vascular territories, including the brain. RIC cycles of ischaemia-reperfusion could be applied during (perconditioning), before (preconditioning, RIPC), or after (postconditioning) the establishment of cerebral ischaemia. The mechanisms underlying this have not been fully characterised but have been shown to involve neural, hormonal, and systemic inflammatory signalling cascades (reviewed in [2]). Although this definition is quite broad, recent findings listed below have indicated the significant involvement of processes associated with the rapid detoxification of glutamate overdose (excitotoxicity) in ischaemia-affected brain regions.

Excitotoxicity is one of the earliest molecular mechanisms of post-ischaemic stroke damage, in which glutamate and N-methyl-D-aspartate receptor (NMDAR) play an essential role [3]. Glutamate is the main excitatory neurotransmitter in the mammalian central nervous system, whereas NMDAR, a glutamate-gated ion channel, controls synaptic plasticity and the initiation of cellular responses in the nervous system. In the pathological condition of ischaemia, the failure of cell metabolism leads to the rapid release of glutamate to the extracellular space of neurons and impaired reuptake. Those conditions ultimately lead to a rapid increase in glutamate levels in the ischaemic regions of the brain, hyperactivation of NMDAR connected to the activation of downstream cell death signals in the cell, and the end spreading of the NMDR activation to the surrounding tissue (reviewed in [4]). Brain tissue excitotoxicity reaches beyond the border of the central nervous system. In the blood, the early stage of post-ischaemic reperfusion reflects an increase in blood glutamate level, ultimately gaining in oxidative stress and affecting the enzyme antioxidant defence [5, 6].

In recent decades, studies on impaired glutamate release and reuptake have become hot topics in the mechanism of neuronal damage in ischaemic stroke and have suggested potential targets for therapeutic interventions. Several studies were focused on extracellular glutamate reduction by its release into the blood. The efflux of glutamate through the BBB is mediated by EAATs located on endothelial cells, where it accumulates until the concentration exceeds that in the plasma, and then is facilitatively transported into blood [7]. Blood glutamate scavenging causes an increased concentration gradient between the brain and blood and provides an increased driving force for glutamate efflux [8]. RIPC as an effective neuroprotective strategy for stroke reflects the close connection to glutamate-mediated excitotoxicity. Decreased neuronal loss in RIPC-treated animals after ischaemia is connected to dramatic changes in brain and blood glutamate levels which reflect the total body oxidative stress. While brain concentrations of glutamate decrease after the RIPC treatment, the blood levels rise. This could indicate that RIPC can mediate accelerated brain-to-blood efflux of glutamate which, in fact, could positively influence neuronal survival in ischaemia-affected brain regions [9, 10]. The efflux of glutamate from the brain to the blood is accelerated naturally based on the concentration gradient [8], but the involvement of other mechanisms intensifying this process should not be excluded. In *ex vivo* conditions, RIPC induces the sequestration of plasma glutamate via EAAT transporters located on the blood cell’s surface and probably contributes to enhanced glutathione synthesis. Therefore, the disbalance of brain/blood glutamate could mediate accelerated efflux to reach homeostasis [11]. However, *in vivo*, the involvement of increased active transport mediated by EAATs transporters in the CNS after RIPC stimulation should be considered.

Therefore, the main idea of this paper was to investigate the involvement of active glutamate transport from the brain to blood after RIPC. To prevent the negative consequences of stroke (such as BBB disruption or inflammation), which may affect glutamate levels, a simulation of stroke-related excitotoxicity was made by the intraventricular injection of glutamate. The EAAT-mediated transport was tracked at the level of the CNS as well as that of circulating blood and blood glutathione monitoring was performed to predict the possible routing of sequestered glutamate. To confirm that EAAT-mediated active transport was a result of RIPC stimulation, the inhibitor of transporter activity was co-administered intraventricularly with the dose of glutamate. Consequently, the following questions about the role of rapid glutamate detoxication in RIPC-mediated neuroprotection have been answered: 1) Does RIPC potentiate active transport of glutamate-mediated by EAATs from the cerebrospinal fluid? 2) Does an overdose of glutamate in the cerebrospinal fluid reflect on the blood level of glutamate? 3) Do EAAT transporters mediate this early rise in blood glutamate concentrations? 4) Does the early increase in blood glutamate influence blood glutathione levels? 5) Do RIPC-mediated changes affect the overall oxidative stress in those animals?

## Methods

The experiments were carried out in accordance with the protocol for animal care approved by the European Communities Council Directive (2010/63/EU) with permission of the State Veterinary and Food Administration of the Slovak Republic (4451/14–221 and 4247/15–221) under the supervision of the ethical council of the Institute of Neurobiology BMC SAS. Every effort was made to minimise animal suffering and reduce the number of animals used. Adult male albino Wistar rats (bred at a certified vivarium of the Institute of Neurobiology BMC SAS originating from Velaz, Czech Republic) weighing 330–350 g were maintained on a 12 h light/dark cycle and given food and water ad libitum. Food was withdrawn one day before surgery.

### Experimental Design

**Fig. 1:**
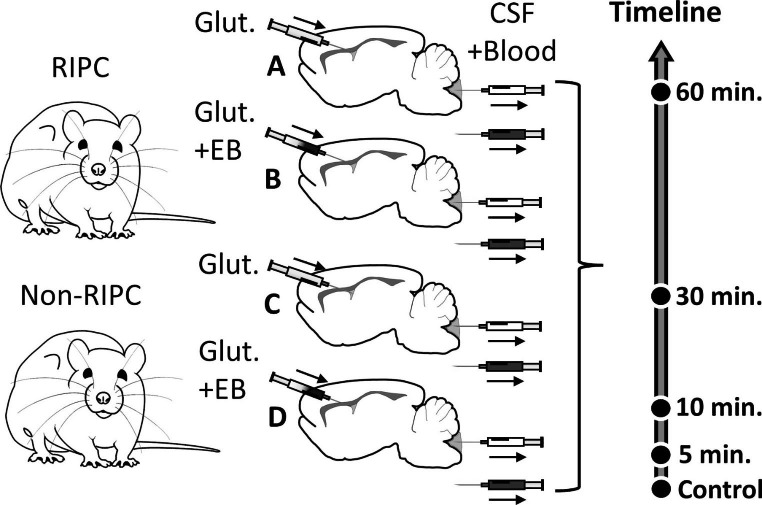
Experimental design. Groups **A** and **B**- animals pre-treated with remote ischaemic conditioning (RIPC, tolerant); **C** and **D** - animals underwent the same procedure as **A** and **B** without a hind limb tourniquet (non-RIPC, non-tolerant). One hour later, glutamate (Glut.) was injected into the right ventricle with **(A, C)** or without **(B, D)** the addition of the EAAT activity inhibitor Evans Blue. The samples of blood and CSF were collected before the procedure (control, **C**) and 5, 10, 30 and 60 min after glutamate administration

Animals were randomly divided into 4 groups. Groups A and B were pre-treated with remote ischaemic conditioning (RIPC, tolerant), while C and D underwent the same procedure without a hind limb tourniquet (non-RIPC, non-tolerant). One hour later, glutamate was injected into the right ventricle with (A, C) or without (B, D) the addition of the EAAT activity inhibitor Evans Blue. The samples of blood and CSF were collected before the procedure (control, C) and 5, 10, 30 and 60 minutes after glutamate administration (Fig. 1).

### Intraventricular Administration of Glutamate

Post-ischaemic glutamate release was imitated by a single dose of glutamate injected into the right lateral ventricle. Animals were anaesthetised with 4% isoflurane in the anaesthetic cage and during surgery were maintained with 1.5% isoflurane. The animal was fixed in the stereotaxic apparatus and the hole was drilled in the skull 1.49 mm right and 0.95 mm posterior to bregma. A single dose of 20 mM glutamate (L-glutamic acid, Sigma-Aldrich) (10 µl) was administered through the hole to the right lateral ventricle by needle (0.95 mm posterior and 1.49 mm lateral to the bregma, 3.6 mm deep). According to Cserr and Berman (1978) [12], the volume of rat CSF is 250 µl, so the injected 20 mM glutamate (or saline as a placebo, 10 µl) in the ventricle was diluted 25-fold to a concentration 800 µM. EAAT activity was inhibited by the intraventricular co-administration of glutamate doses and Evans Blue in a final concentration of 220nM. Evans Blue (EB) is a potent competitive inhibitor of vesicular glutamate uptake. EB is a structural analog of glutamate that shows severalfold higher affinity to the glutamate transporter- while Evans Blue is efficient for competitive inhibition of glutamate transport with a value of inhibitor constant Ki in the nanomolar scale, the Km value of the glutamate uptake has been determined to be millimolar [13, 14].

### RIPC Treatment and Blood/Liquor Samples Collection

Right hind-limb ischaemia was induced by placing an elastic rubber band tourniquet on the proximal part of the limb for 5 min, followed by a 5-min period of reperfusion according to [15] (tolerant, RIPC). The procedure was performed under light isoflurane anaesthesia (0.5%) in three cycles 1 h before blood sampling. Non-tolerant (non-RIPC) animals underwent the same procedure without the tourniquet application. The samples of blood/liquor were collected in rated intervals (see experimental design) from the jugular vein or cisterna magna respectively, via the polyethylene tubing (BTPE10, Instech, USA) applied before the glutamate application.

### Determination of Blood Glutamate and Glutathione

First, samples of blood/liquor were deproteinised by adding ice-cold 1M perchloric acid (PCA, 1:1 v/v, 10 min on ice) and centrifuged (12,000 rpm, 10 min, 4°C). The supernatant was collected and stored at -80°C for subsequent analysis. In liquor samples of the group where Evans Blue inhibition was performed, BSA in final concentration 0.5 mg/ml was applied for 10 min before deproteinisation to bind the rest of the dye.

The glutamate concentration of samples was measured by a modified enzymatic-fluorometric method [5] based on [16]. The detection of NADH fluorescence resulting from the reaction of glutamate in precipitated samples and NAD^+^ catalysed by glutamate dehydrogenase was used to determine glutamate content. The glutamate concentration is directly proportional to the concentration of NADH in a reaction. Briefly, 10 µl of supernatant was pipetted into a black 96-well plate, and 190 µl of reaction buffer (0.25 M hydrazine hydrate/0.3 M glycine buffer, pH 8.6) containing 200 nM NAD^+^ and 15 U of glutamate dehydrogenase was added. After 30 min of incubation at RT, the fluorescence intensity of the final product (NADH) was read on a Synergy™ 2 Multi-Mode Microplate Reader (BioTek) at 460 nm with an excitation wavelength of 360 nm. The concentration of glutamate in all samples was recalculated per litre of total blood (µmol/l of blood) and CSF (µmol/l of CSF). To determine glutamate changes in liquor/blood 10 min after its intravenous application (rapid phase, Fig. 5), the value was expressed as a percentage and the value in the 5th min was set as 100%. All chemicals used for glutamate concentration measurements were purchased from Fluka.

Total glutathione content in the samples of whole blood was measured using the colorimetric method from [17] with minor modifications. Samples of precipitated blood were incubated with 10 mM Ellman’s reagent in 500 mM TRIS buffer (pH 8.2) with 10 mM EDTA (10 min, RT). After incubation, the absorbance at 415 nm was measured, and the total glutathione concentration was expressed per litre of blood. Glutathione (scale from 0 to 15 nM) was used as a calibrator for the assay.

### Determination of Oxidative Stress

#### Comet Assay

The comet assay was carried out under alkaline conditions as reported by [18], with minor modifications to evaluate DNA strand breaks in peripheral lymphocytes. Microscope slides were pre-coated with 1% normal-melting-point agarose in PBS (pH 7.4) and allowed to dry on a flat surface at room temperature (RT). A lymphocyte suspension was mixed with 1% low-melting-point agarose in PBS (pH 7.4), spread over agarose pre-coated slides, covered by coverslips and allowed to dry in a refrigerator for 20 min. When the agarose gel solidified, the coverslips were removed and the slides were immersed in lysis solution (2.5 mol/L NaCl, 100 mmol/L Na_2_EDTA, 10 mmol/L Tris, 1% Triton X-100, 10% DMSO, pH 10) for 1 h at 4°C. The slides were transferred to alkaline electrophoresis buffer (5 mol/L NaOH, 200 mmol/L Na2EDTA, pH 13) for 20 min at 4°C and then electrophoresis was carried out at 25V for 25 min at 4°C. After electrophoresis, the slides were neutralised in a neutralisation buffer solution (400 mmol/L Tris, pH 7.5) for 15 min at 4°C. After air-drying, DNA was visualised using SYBR Green and images were captured using a fluorescence microscope (Olympus BX51, ex.f.485, em.f.520 nm) equipped with a camera (Olympus DP50). Images were analysed using the Comet ScoreTM v1.5 image analysis system (TriTek Corp., USA). DNA damage was assessed by the parameter “% DNA in tail” (100% of cell fluorescence intensity minus intensity of the head % DNA).

#### Superoxide Dismutase Antioxidant Defence

Superoxide dismutase (SOD) activity was measured by a modification of the protocol described by [19]. The standard assay substrate mixture contained 1 M xanthine (Sigma), 0.1 M EDTA, 5.6 x 10^− 2^ M NBT (p-nitrotetrazolium blue grade III, Sigma-Aldrich, Steiheim, Germany) and 1 M BSA (bovine serum albumin, Fluka) in 0.1 M sodium phosphate (pH 7.8). The assay uses xanthine-xanthine oxidase as a superoxide generator to prevent NBT reduction by superoxide. The absorbance of NBT reduced to blue formazan by superoxide at 560 nm at room temperature was measured. SOD in the reaction prevents the synthesis of blue formazan. In blood cells, one unit of SOD activity was defined as the amount that reduced the absorbance change by 50%. The SOD activity was expressed as kU per milliliter of blood (kU/ml of blood).

### Statistical Analysis

Data were analysed and plotted using GraphPad Prism (Graph-Pad Software, San Diego, California, USA). Statistical analysis was performed using one-way and two-way ANOVA, followed by the Dunnett post hoc test. The criterion for statistical significance was p < 0.05.

## Results

### RIPC Potentiates a Rapid Decrease of Glutamate from Cerebrospinal Fluid

**Fig. 2:**
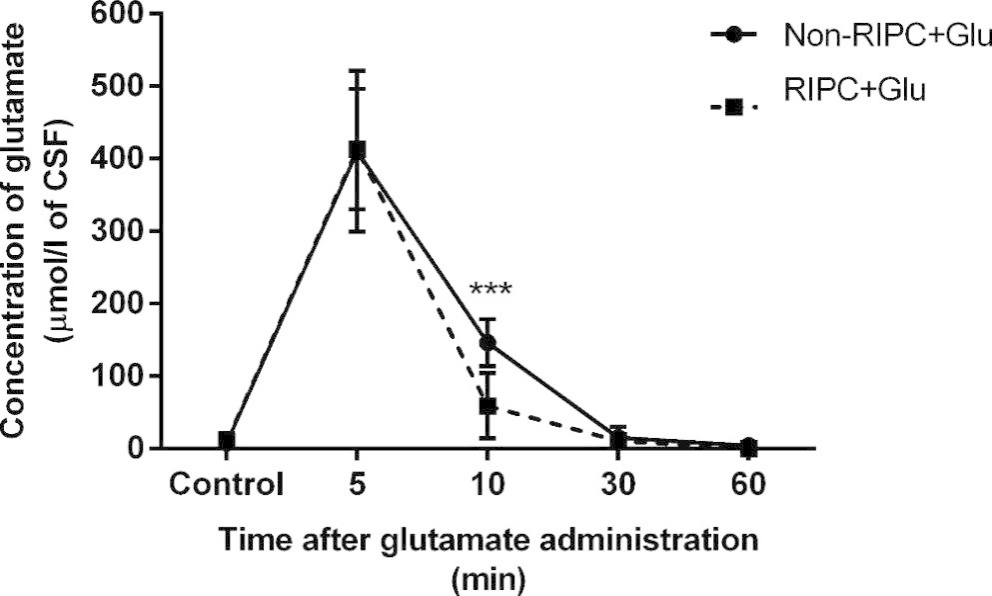
The course of detoxication of glutamate from cerebrospinal fluid after its intraventricular administration in animals with and without remote ischaemic preconditioning. Mean ± SD (***p < 0.001 Non-RIPC + Glu compared to RIPC + Glu). Non-RIPC + Glu- animals without remote ischaemic preconditioning followed by the intraventricular administration of glutamate, RIPC + Glu- animals with remote ischaemic preconditioning followed by the intraventricular administration of glutamate

The effect of RIPC on glutamate overdose in CSF was exhibited especially in the early stage after an introduction by its rapid elimination. Five minutes after the intraventricular injection of glutamate in both RIPC treated and untreated rats, it reached a concentration of about 400 µmol/L (Fig. 2). Five minutes later, i.e. 10 minutes after administration, its level significantly decreased in RIPC-treated animals (59.2 ± 45 µmol/L) compared to untreated counterparts (146.4 ± 32.5 µmol/L). RIPC treatment in this group reduced the CSF glutamate level by around 87% at this time. The amino acid level was normalised and reached the control value in both groups 30 minutes after application, which suggests that RIPC has an effect in the early stages of glutamate detoxication.

### RIPC Mediates Increase in the Blood Level of Glutamate After its Intraventricular Administration

**Fig. 3:**
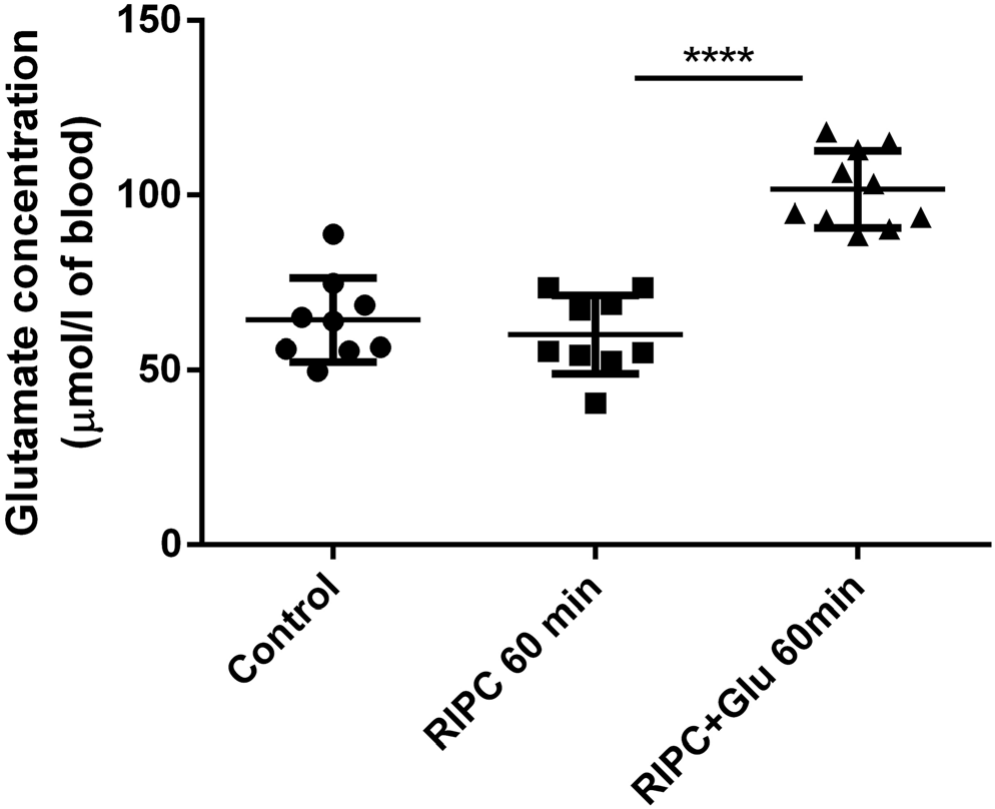
Blood level of glutamate in RIPC-treated animals one hour after the intraventricular application of glutamate/placebo. All animals underwent the same surgical procedure of intraventricular placebo/glutamate administration with or without tourniquet pre-treatment. The blood was collected one hour after the procedure. Control- animal subjected to intraventricular placebo administration without RIPC. RIPC 60 min- animal subjected to intraventricular placebo administration with RIPC. RIPC + Glu 60 min- animal subjected to intraventricular glutamate administration with RIPC. RIPC- remote ischaemic preconditioning; Glu- glutamate; ****p < 0.0001

In the first stage of experiments, the application of RIPC by itself was shown to have no effect on the blood level of glutamate; therefore, skeletal muscle ischaemia does not influence blood glutamate levels one hour after treatment. Glutamate concentrations increase only in the group of animals where RIPC was followed by the intraventricular injection of glutamate (Fig. 3), which suggests a direct link between CSF glutamate levels and RIPC-mediated blood glutamate elevation.

**Fig. 4:**
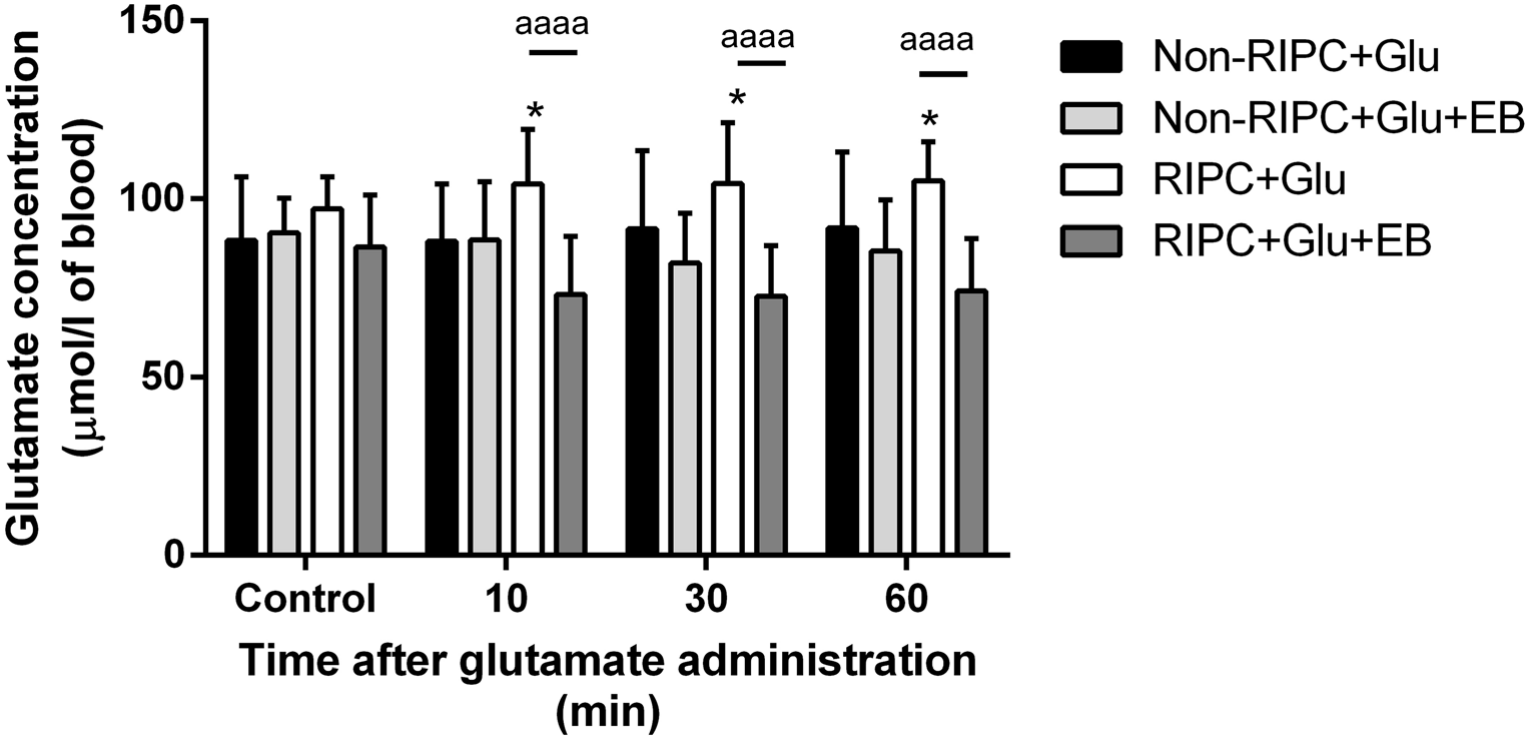
Course of blood glutamate level after the intraventricular administration of glutamate and the inhibition of EAAT activity in brain parenchyma. Mean ± SD (*p < 0.05 Non-RIPC + Glu compared to RIPC + Glu; ^aaaa^p < 0.0001 RIPC + Glu compared to RIPC + Glu + EB). Non-RIPC + Glu- animals without remote ischaemic preconditioning followed by intraventricular administration of glutamate, RIPC + Glu- animals with remote ischaemic preconditioning followed by the intraventricular administration of glutamate; EB- intraventricular co-administration of Evans Blue

To investigate whether the RIPC-mediated glutamate elevation in injected animals is related to CSF efflux, the EAAT inhibitor (Evans Blue) was intraventricularly co-administered with glutamate to inhibit brain parenchyma EAATs. In contrast to non-treated rats (88 µmol/L of blood), RIPC mediates a rapid increase in blood glutamate even 10 min after its administration (104 µmol/L of blood, elevation by about 20% compares to RIPC untreated group) and remains elevated for up to one hour. The co-administration of the EAAT inhibitor Evans Blue to the CSF diminished the effect of RIPC pre-treatment and significantly reduced the blood level of glutamate for up to one hour. This effect was not shown in RIPC-untreated rats suggesting the RIPC-mediated increase in EAAT activity in brain parenchyma (Fig. 4).

### Early Rise of Glutamate Concentration in the Blood Circulation of RIPC-Treated Animals After its Intraventricular Administration is Related to EAATs-Mediated Transport

**Fig. 5:**
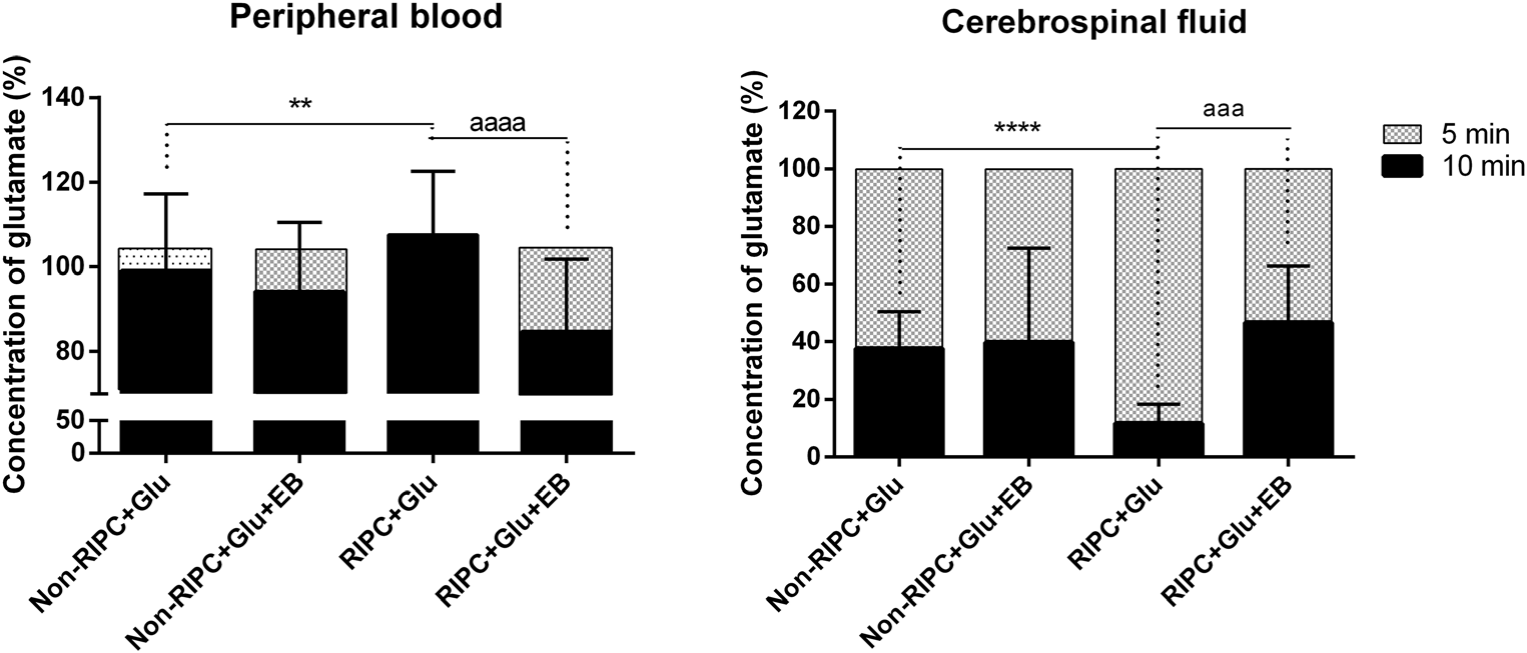
Percentage changes in glutamate levels in the cerebrospinal fluid and peripheral blood ten minutes after its intraventricular injection, the effect of the inhibition of EAATs activity in brain parenchyma. CSF glutamate concentration five minutes after the injection was set to 100%. Control value of blood glutamate level was set to 100%. Mean ± SD (**p < 0.01, ****p < 0.0001 Non-RIPC + Glu compared to RIPC + Glu; ^aaa^ p< 0.001, ^aaaa^p < 0.0001 RIPC + Glu compared to RIPC + Glu + EB). Non-RIPC + Glu- animals without remote ischaemic preconditioning followed by the intraventricular administration of glutamate, RIPC + Glu- animals with remote ischaemic preconditioning followed by the intraventricular administration of glutamate; EB- intraventricular co-administration of Evans Blue

As shown in Figs. 2 and 4, the major decrease in glutamate in the CSF of RIPC-treated rats and its significant rise in the blood was detected 10 minutes after injection. This suggests that RIPC stimulated the EAAT-mediated transport of glutamate from the CSF to the blood in circulation. In contrast to RIPC-untreated animals (drop by about 62%), RIPC stimulation led to CSF glutamate decrease by about 88.3% from the base maximal level measured 5 minutes after the glutamate injection. In the blood of the RIPC-treated group, the glutamate concentration increased significantly to 107.5 ± 15.11%. The inhibition of EAAT-mediated transport in the brain parenchyma did not influence the RIPC-untreated group, but dramatically increased glutamate concentration in the CSF (to 46.6 ± 20%) and significantly reduced its blood level to 84.8 ± 17%. Taken together, RIPC stimulated EAAT activity to the rapid efflux of glutamate overdose in CSF to blood circulation. The results were expressed as a percentage of control values (Fig. 5).

### RIPC Potentiates the Increase in Blood Glutathione Concentrations

**Fig. 6:**
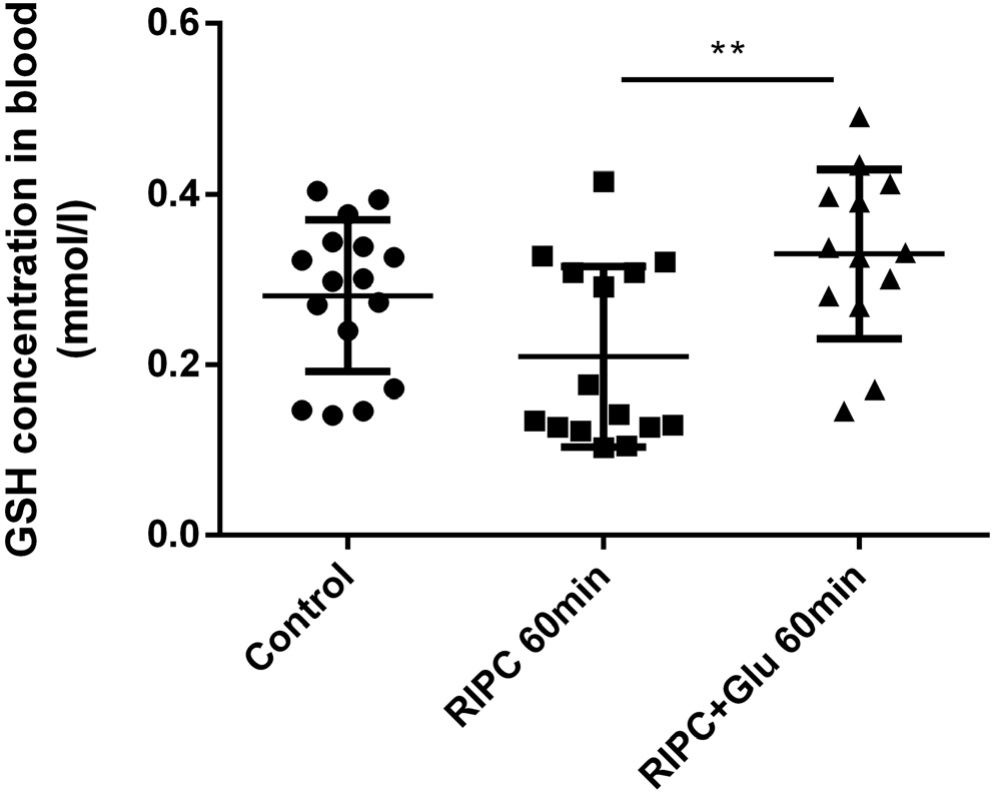
Blood levels of glutathione in RIPC-treated animals one hour after the intraventricular application of glutamate/placebo. All animals underwent the same surgical procedure of intraventricular placebo/glutamate administration with or without tourniquet pre-treatment. The blood was collected one hour after the procedure. Control- animal subjected to intraventricular placebo administration without RIPC. RIPC 60 min- animal subjected to intraventricular placebo administration with RIPC. RIPC + Glu 60 min - animal subjected to intraventricular glutamate administration with RIPC. RIPC- remote ischaemic preconditioning, GSH- glutathione; ^**^ p < 0.01

**Fig. 7:**
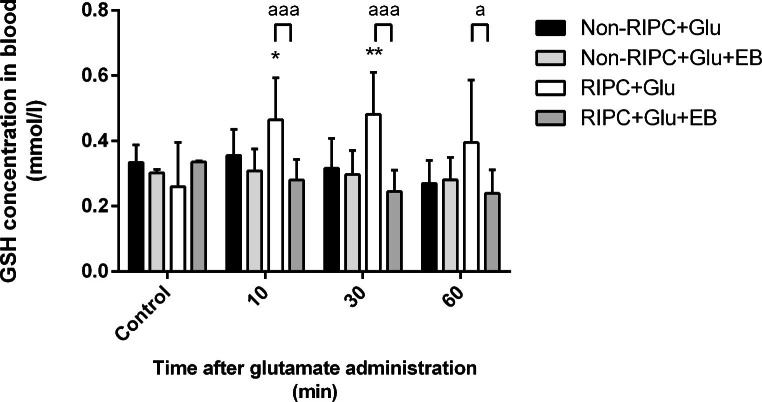
Course of blood glutathione levels after the intraventricular administration of glutamate and inhibition of EAATs activity in brain parenchyma. Results are expressed as a percentage of control. Mean ± SD (*p < 0.05, **p  < 0.01 Non-RIPC + Glu compared to RIPC + Glu; ^a^p < 0.05, ^aaa^p < 0.001 RIPC + Glu compared to RIPC + Glu + EB). Non-RIPC + Glu- animals without remote ischaemic preconditioning followed by the intraventricular administration of glutamate, RIPC + Glu- animals with remote ischaemic preconditioning followed by the intraventricular administration of glutamate; EB- intraventricular co-administration of Evans Blue

The blood level of glutathione measured one hour after the RIPC application did not differ compared to the control value, i.e. RIPC does not have any effect on its changes. However, RIPC in combination with subsequent intraventricular glutamate injection led to the escalation of blood glutathione by about 55% (to 0.326 mmol/l of blood) compared to individuals who have undergone only the RIPC procedure (0.209 mmol/l of blood) (Fig. 6). In the context of the previous result dealing with the blood glutamate elevation in this group, it suggests the connection between the blood glutamate and glutathione levels and RIPC-mediated glutamate efflux from the CSF. To confirm this hypothesis, the blood glutathione level was also measured in rats with blocked EAAT activity in the brain parenchyma (animals followed glutamate and Evans Blue co-administration into the brain ventricle). However, there were no significant changes in rats untreated by RIPC after glutamate injection up to the first hour when the EAAT activity was blocked, the RIPC-treated group reflected a dramatic shift. The level of glutathione over 60 minutes dropped from 0.465 ± 0.12–0.395 ± 0.19 mmol/l to 0.281 ± 0.06–0.239 ± 0.07 mmol/l of blood due to EAAT inhibition (Fig. 7). Taken together, because there were no changes after EAAT blocking by EB in a group of RIPC untreated rats and a rapid drop of glutathione in the RIPC + Glu + EB group, the direct and rapid influence of the EAAT-mediated transport of glutamate from the CSF to the blood on blood glutathione concentrations after remote ischaemic conditioning could be expected.

### RIPC Mediates the Reduction of Oxidative Stress in Animals After Intraventricular Glutamate Administration

**Fig. 8:**
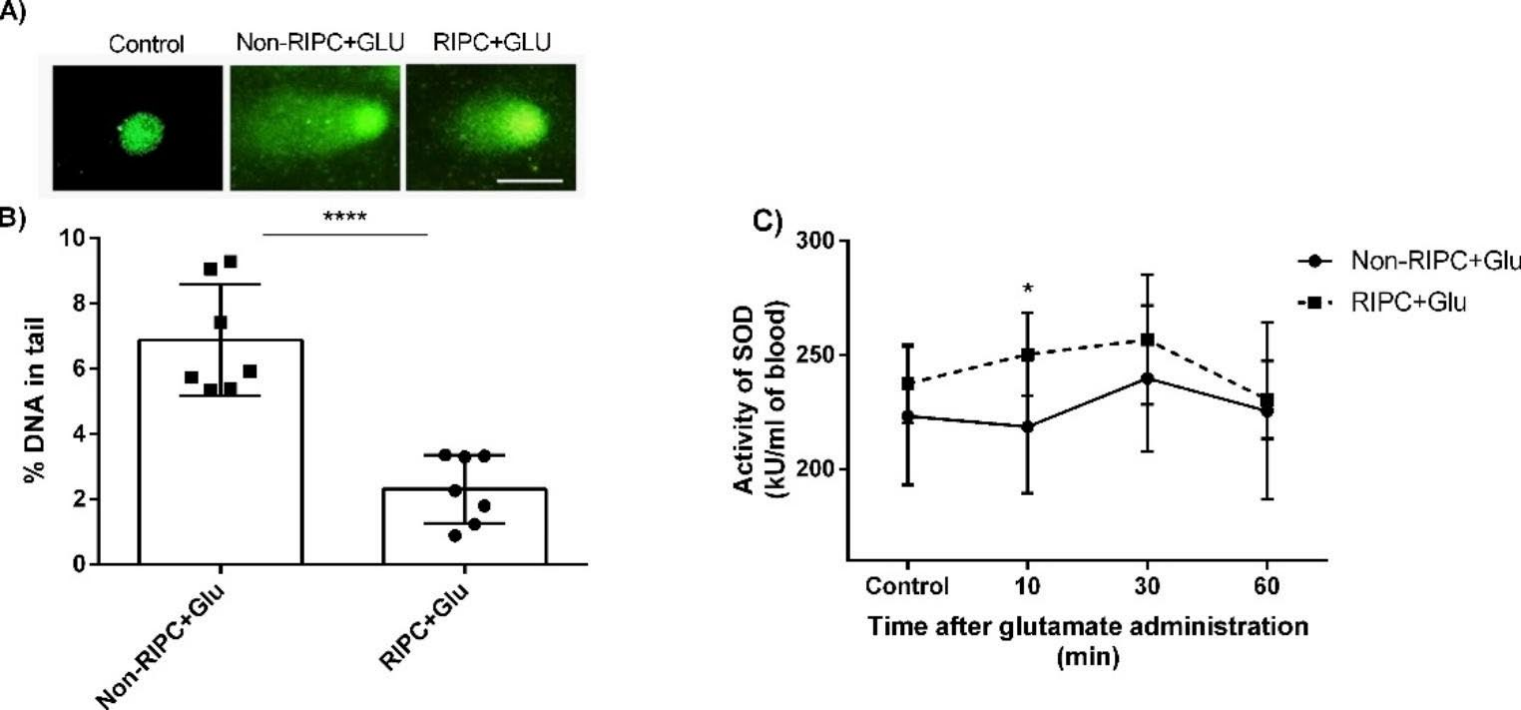
Oxidative damage of peripheral lymphocytes. **(A)** Representative figure of stained lymphocyte DNA in different stages of damage characterised by the percentage amount of fragmented DNA in the tail of the comet. **(B)** Comparison of lymphocyte DNA damage and **(C)** activity of superoxide dismutase (SOD) in the blood cells between groups of RIPC- treated and untreated animals after the intraventricular injection of glutamate. Mean ± SD (*p < 0.05, ****p < 0.0001 Non-RIPC + Glu compared to RIPC + Glu). Non-RIPC + Glu- animals without remote ischaemic preconditioning followed by the intraventricular administration of glutamate. RIPC + Glu- animals with remote ischaemic preconditioning followed by the intraventricular administration of glutamate

To define the extent of the oxidative stress in RIPC-treated animals, the rate of DNA damage of peripheral lymphocytes and the activity of an antioxidant enzyme superoxide dismutase was determined. Remote ischaemic preconditioning significantly reduced oxidative stress, defined as the percentage of DNA in the tail of the lymphocyte comets to 2.3 ± 1% DNA in the tail, i.e. by about 66.5% less compared to untreated rats after glutamate exposure (Fig. 8A, B). Similarly, the activity of the antioxidant enzyme SOD is reduced in animals after RIPC and glutamate injection (Fig. 8C). However, the activity in those animals was significantly higher during the first hour compared to their untreated counterparts 10 minutes after the intraventricular injection (by about 14.5%).

## Discussion

Glutamate-mediated excitotoxicity is one of the earliest, important mechanisms leading to brain tissue damage after the metabolic failure caused by ischaemia. The main characteristic of excitotoxicity is overactivation of NMDAR glutamate receptors and its downstream cellular signals. It is logical to assume that NMDAR stimulation is a central step in post-stroke damage and the interruption at this point will lead to massive neuroprotection. Unfortunately, direct NMDAR blockers have failed in the treatment of excitotoxic damage after ischaemia [20, 21], probably due to the dual roles of NMDARs in neuronal survival and death (reviewed in [20]). Because the decrease in extracellular glutamate seems to be related to improved neuronal survival after ischaemia [9, 10, 22], our investigation has focused on the mechanisms of the brain to blood efflux for restoring glutamate homeostasis.

Remote ischaemic conditioning has shown strong neuroprotective potential against ischaemia-mediated damage of brain tissue (reviewed in [2]). When focusing on glutamate, RIC applied before or after ischaemia significantly affects extracellularly released glutamate as well as glutamate in the circulation and seems to be related to RIC-mediated neuroprotection [9, 10, 22]. During acute ischaemia, extracellular glutamate levels rise sharply within a short period [23]. A crucial component of extracellular glutamate increase during ischaemia originates from the neurons/glia combined with a rapid drop in the expression of the glutamate transporters EAAT1 or EAAT2 [24], and from immune cells which have released glutamate in the damaged tissue [25, 26]. After reperfusion, glutamate can be partially removed from the ischaemic tissue by reconstructed blood flow [27], which could be associated with elevated levels of blood glutamate during the post-ischaemic reperfusion [5, 22, 28]. However, hypoxic conditions stimulate several immune cells in circulation to the release glutamate and consequently accelerates its blood concentration [29].

We have shown, that the RIPC significantly accelerates the rapid clearance of glutamate from the CSF early after its intraventricular administration. At the same time, the blood glutamate level increased over the baseline. Because the effect of RIPC-treatment on blood glutamate elevation via immune cell activation was contradicted, the peripheral glutamate rise after RIPC originated from its intraventricular overdose. Blood elevation of glutamate depends on the activity of parenchymal EAATs, the blocking of which completely reduced its release into the circulation, resulting in a glutamate decrease, even under normal concentrations. Malina et al. [30] provided evidence that the EAATs transporters presented on endothelial cells constituting the BBB can be involved in the efflux mode via the selective removal of glutamate. Glial cells lining the BBB have an active role in the efflux process by taking up glutamate and releasing it in a close vicinity to the endothelium and accelerating its efflux to the blood. After RIPC, the glutamate transporter EAAT2 was found to be significantly upregulated up to three days after reperfusion, supporting the assumption that the upregulation of EAAT2 expression may play an important role in RIPC-mediated neuroprotection [31]. However, our results confirm the role of brain parenchyma EAATs activity/upregulation in glutamate efflux from CSF to blood after RIPC treatment, the EAAT subtypes contribution and their localisation within the CNS have been the subject of our ongoing study.

The accumulation of peripheral glutamate may lead to the elevated production of ROS and subsequently to increased oxidative stress, which could represent an unfavorable factor for the prognosis of cerebral ischaemia [5]. Comet assay has been established as an effective method for determining the oxidative stress of the organism. Increased production of ROS is considered the main reason for DNA damage of peripheral lymphocytes while the cellular antioxidant system is the main tool for its prevention [32]. Glutamate receptors, namely NMDA receptors, have been found to cause the increase of ROS production in the whole blood as well as in lymphocytes, however in a dose-dependent manner. While the 500 µmol NMDA concentration in the blood causes ROS increase compares to the control, a lower dose of 50 µmol NMDA cause more than three times higher ROS production [33]. Our previous work confirmed the positive correlation between peripheral lymphocyte DNA damage/ blood cell enzyme antioxidant activity of SOD and the elevated blood level of glutamate after brain ischaemia [5]. In the paper, the blood glutamate top after the ischaemia (elevation by about 40 µmol/l over the control) correlated to the maximum DNA damage and to elevated SOD activity. In the experiment presented in the manuscript, the maximal blood glutamate level of the RIPC untreated group was up to 4 µmol/l over the control, and up to 10 µmol/l over the control in RIPC-treated rats. Moreover, the activity of the SOD enzyme (as well as the GSH level) was higher in the blood of RIPC-treated rats. Taking the above-mentioned arguments into account, the differences in the balance of ROS production and antioxidant defense between experimental groups could result in significant lover oxidative stress following the RIPC treatment.

Concentrating on the peripheral blood and the next glutamate use, glutamate transporters could also be found to be expressed in blood cells. EAATs were detected in platelets and white blood cells, as well as in erythrocytes [11, 34–36], although at a lower density compared to the brain. Interestingly, the transport activity of blood cell transporters could be altered in some neuropsychiatric disorders [37] or following chemical stimulation [38]. The fate of the scavenged glutamate in those cells seems to be specific to the cell type. In erythrocytes, the upregulated uptake of glutamate via activated EAAT transporters exposed to arsenite could serve as a source for the synthesis of the antioxidant tripeptide glutathione [38], an important free-radical scavenger that is present in all cells.

The results presented in this paper confirmed the impact of RIPC treatment on glutathione production and the elimination of oxidative stress on peripheral blood cells. Blood levels of glutathione increased significantly during the first hour after the intraventricular injection of glutamate in those rats, which was reflected in a notable reduction of the oxidative damage of peripheral lymphocytes as well as in the activation of the cellular antioxidant enzyme SOD (discussed above). Moreover, the inhibition of EAAT-transporters expressed on brain parenchyma manifested intraventricularly injected glutamate as a relevant source of glutathione for circulating cells. Those outcomes are in line with our previous observation where the response of the blood cells derived from RIPC-subjected animals to glutamate overdose *ex vivo* was studied [11]. In this study, we confirmed the existence of EAAT transporters on the membranes of certain blood cell populations. Moreover, transporters could be affected by RIPC in the manner of activity/quantity in the presence of free glutamate that reflects on the overall glutathione concentration. Taking into account the consequences of both *in vivo* and *ex vivo* approaches it could be presumed that EAAT transporters could be the key effector of RIPC stimulation. However, the hypothesis should be carefully verified by future studies.

## Conclusion

We can conclude that RIPC could partially affect the mechanisms of glutamate homeostasis. RIPC mediates the efflux of glutamate from the CSF to the blood in the very early stages of intoxication and ensures the rapid clearance of abnormal levels of glutamate in the CSF to normal conditions. This process is provided by EAAT glutamate transporters of brain parenchyma, the activity of which is significantly elevated after RIPC treatment. As a consequence, the blood level of glutamate rises in a moment. Excess blood levels of glutamate serve as a relevant source of antioxidant glutathione for circulating cells, the concentration of which is growing in conditioned animals. All of those improvements in the processes of glutamate homeostasis recovery reflect the improvement of oxidative stress. With respect to our previous findings, glutamate excess detoxication (probably related to EAATs) could be one of the key mechanisms of RIPC-mediated neuroprotection.

## Data Availability

Data are available from the corresponding author on request.
